# A facile and efficient synthesis approach of salidroside esters by whole-cell biocatalysts in organic solvents

**DOI:** 10.3389/fbioe.2022.1051117

**Published:** 2022-11-24

**Authors:** Rongling Yang, Yu Wang, Xiangjie Zhao, Zheng Tong, Qianlin Zhu, Xiaoxi He, Zhaoyu Wang, Hongzhen Luo, Fang Fang

**Affiliations:** School of Life Science and Food Engineering, Huaiyin Institute of Technology, Huaian, China

**Keywords:** salidroside esters, whole-cells, biocatalysis, acyl donor specificity, organic solvents

## Abstract

Salidroside, the main bioactive compound isolated from the plant source of *Rhodiola rosea* L, possesses broad-spectrum pharmacological activities, but suffers from the low cell membranes permeability and alimentary absorption due to its high polarity. Therefore, a whole-cell catalytic strategy for the synthesis of salidroside esters was explored to improve its lipophilicity. The results showed that *Aspergillus oryzae* demonstrated the highest biocatalytic activity among the microbial strains tested. For the synthesis of salidroside caprylate, the optimum conditions of reaction medium, *Aspergillus oryzae* amount, molar ratio of vinyl caprylate to salidroside and reaction temperature were acetone, 30 mg/ml, 10°C and 40°C, respectively. Under these conditions, the initial reaction rate was 15.36 mM/h, and substrate conversion and regioselectivity all reached 99%. Moreover, the results indicated that although various 6′-monoesters derivatives of salidroside were exclusively obtained with excellent conversions (96%–99%), the reaction rate varied greatly with different chain-length acyl donors. This study details an efficient and cost-effective biocatalytic approach for the synthesis of salidroside esters by using *Aspergillus oryzae* as a catalyst for the first time. Considering the whole cell catalytic efficiency and operational stability, this strategy may provide a new opportunity to develop green industrial processes production for ester derivatives of salidroside and its analogues.

## 1 Introduction

Salidroside (4-hydroxyphenethyl-β-D-glucopyranoside) is the main bioactive ingredient isolated from *Rhodiola rosea* L that has been used as a traditional Chinese medicine for a long time (Zhang et al., 2018). Recent studies confirmed that salidroside possesses broad-spectrum pharmacological activities including anti-hypoxic, anti-fatigue, anti-inflammation, anti-cancer, anti-convulsion, as well as protecting the cardiovascular system, exerting neuroprotective effects, improving glucose and lipid metabolism (Hu et al., 2010; Xing et al., 2018; Wu et al., 2020; Yang L. M. et al., 2020; Qi et al., 2021; Qian et al., 2021; Song et al., 2021; Wu et al., 2021). However, the multiple hydroxyl groups of salidroside make it highly polar, which results in the low cell membranes permeability and alimentary absorption (Liang et al., 2021). Various attempts have been made to improve the bioavailability of phenolic glycosides by modifying their structure to balance their lipophilicity and hydrophilicity. Many studies have found that acylation modification of natural glycosides not only improve their bioavailability but also could increase their pharmacological properties, making them suitable candidates for prodrug development (Chyba et al., 2016; Chen et al., 2019; Yang R. L. et al., 2019; Liu et al., 2020; Hao et al., 2021; Yang et al., 2021; Zhang P. L. et al., 2021). For example, tri-acetylated phloridzin displayed significantly higher anti-proliferative activity against human HepG2 cancer cells than phloridzin, while exhibited moderate to minimal adverse-effects on LO-2 normal hepatic cells (Chen et al., 2019). The intracellular antioxidant activities of acylated cyanidin-3-glucoside were significantly enhanced relative to cyanidin-3-glucoside due to their increased lipophilicity (Zhang X. M. et al., 2021).

Salidroside is a kind of polyhydroxy natural glycosides carrying several hydroxyl groups with similar chemical activity. The biocatalytic acylation of polyhydroxy compounds based on the whole cells and isolated enzymes has become a promising approach due to the mild reaction conditions, high regioselectivity and environmental friendliness (Yu et al., 2008; Chen et al., 2013; Li G. Y. et al., 2018; Sugai et al., 2018; Jeandet et al., 2020; Hao et al., 2021). Salidroside was successfully acylated with aliphatic acids by Novozyme 435, an expensive commercial immobilized lipase (Yu et al., 2008). Compared to purified or immobilized enzymes, employing whole cells biocatalysts can significantly reduce costs by circumventing cell lysis and enzyme purification (Wachtmeister and Rother, 2016). In addition, residual cell wall compounds also have a protective effect on stationary or dead cells, thus enabling them to catalyze reactions in unconventional (non-aqueous) reaction media (Xu et al., 2016; Xin et al., 2017). Hence, the whole cell biocatalysts offered a huge potential in the field of non-aqueous reaction media. Recently, the whole cell biocatalysts displayed similar or better catalytic activity than commercial lipases in the synthesis of glycoside ester derivatives (Yang R. L. et al., 2020; Hao et al., 2021). It is especially critical to identify suitable microbial strains as whole-cell catalysts to catalyze specific natural products, because different microbial cells have different substrate specificities. According to our knowledge, there is no report on the synthesis of salidroside fatty acid ester derivatives catalyzed by the whole cell biocatalysts. Considering the application potential of salidroside esters in pharmaceuticals, the efficient synthesis catalyzed by microbial whole-cells in non-aqueous solvents was firstly investigated. And eight salidroside ester derivatives with different lengths of aliphatic chains were synthesized and structurally identified.

## 2 Materials and methods

### 2.1 Materials

Salidroside was obtained from Aladdin (Shanghai, China). The fatty acid vinyl esters as acyl donors were provided by Tokyo Chemical Industry, TCI (Shanghai) Development Co., Ltd. All microbial strains (*Pseudomonas fluorescens*, *Pseudomonas stutzeri*, *Pseudomonas aeruginosa*, *Aspergillus niger*, *Aspergillus oryzae* and *Rhizopus oryzae*) were obtained from Guangdong Institute of Microbiology (Guangzhou, China). Other chemicals were of analytical grade.

### 2.2 Preparation of whole-cell biocatalysts

Bacterial and fungal strains were cultivated as described previously (Yang L. M. et al., 2020). The bacterial strains were activated in the medium contained 1% sucrose, 1% beef extract, 1% peptone, 0.5% NaCl, 0.5% K_2_HPO_4_ and 0.02% MgSO_4_ 7H_2_O. The fungal strains were activated on potato dextrose agar (PDA) medium. Then, the activated bacterial suspension and fungal spore suspension were inoculated into the same fermentation broth respectively, which contained 0.1% soybean oil, 0.2% tryptone, 0.5% (NH_4_)_2_SO_4_ and 0.02% MgSO_4_ 7H_2_O. After cultivation, the cells were collected by filtration or freeze-centrifugation and freeze-dried for 24 h.

### 2.3 Synthesis of salidroside esters

In a typical experiment, salidroside (20 mM), vinyl caprylate and whole-cell catalyst preparation were added in organic solvents and incubated at 200 rpm. The samples were collected at predetermined time intervals and detected by high-performance liquid chromatography (HPLC). The control experiments without whole-cell catalysts displayed no acylation action. The conversion (C) was measured as the ratio of transformed to initial salidroside. The initial reaction rate (V_0_) refers to the substrate consumption per unit time in the initial stage, in which the substrate concentration decreased linearly with the reaction time. The experiments were carried out in triplicates.

### 2.4 Operational stability of whole-cell biocatalysts

The operational stability of *Aspergillus oryzae* cells during the batch reaction was investigated. The reaction was carried out in anhydrous acetone containing 20 mM salidroside, 200 mM vinyl caprylate, 20 mg/ml whole-cell biocatalysts for 12 h at 40°C and 200 rpm. After each batch synthetic reaction, whole-cell biocatalysts were separated by filtration, washed with reaction medium and utilized in the next fresh reaction.

### 2.5 HPLC analysis

All samples were analyzed by RP-HPLC on an Agilent Zorbax Eclipse Plus C18 column (4.6 mm × 250 mm, 5 μm) using Shimadzu LC-200C pump with the DAD detector at 275 nm. The flow rate was 1.0 ml/min. The mobile phase contained a mixture of methanol and water at a 1.0 ml/min flow rate. The volumetric ratio of methanol to water and the retention times for its ester derivatives were 60/40 and 3.018 min (salidroside-6'-acetate), 60/40 and 4.278 min (salidroside-6'-butyrate), 80/20 and 3.265 min (salidroside-6'-hexanoate), 80/20 and 4.355 min (salidroside-6'-caprylate), 80/20 and 6.782 min (salidroside-6'-decanoate), 80/20 and 6.621 min (salidroside-6'-undecenoate), 90/10 and 4.760 min (salidroside-6'-laurate), 90/10 and 6.623 min (salidroside-6'-myristate), 90/10 and 9.981 min (salidroside-6'-palmitate), respectively.

### 2.6 Structure characterization of the products

All the synthetic products were purified through flash column chromatography using a mixture of ethyl acetate and petroleum ether as the mobile phase. The structural identification of the ester derivatives was determined by ^13^C NMR and ^1^H NMR (Bruker DRX-400 NMR Spectrometer) at 100 MHz and 400 MHz, respectively, with DMSO-d_6_ being the solvent. All the NMR spectroscopic results are shown in the supplementary information.

## 3 Results and discussion

### 3.1 Strains screening for salidroside ester synthesis

Several lipase-producing strains including bacteria and fungi were screened as the biocatalysts for the synthesis of salidroside caprylate (Table 1). Results revealed that all the tested strains showed different catalytic activities. *Aspergillus oryzae* and *Pseudomonas aeruginosa* demonstrate significant outstanding catalytic activity, affording 95.72% and 93.64% conversion after 24 h, respectively, and both gave substantially superior result to those obtained with the other strains that were tested. The reaction catalyzed by *Rhizopus oryzae*, *Pseudomonas fluorescens* and *Pseudomonas stutzeri* proceeded with 20.83%, 11.53% and 9.47 conversion rate after 24 h, respectively. These results indicated that catalytic properties of different microorganisms in the same induction medium were different, which was consistent with previous studies (Xin et al., 2017; Hao et al., 2021). Interestingly, in the regioselective synthesis of helicid aliphatic esters, *Pseudomonas aeruginosa* exhibited excellent catalytic performance, while *Aspergillus oryzae* displayed no catalytic activity (Yang W. et al., 2019). In the synthesis of esculin esters, *Pseudomonas stutzeri* demonstrated the highest catalytic activity (Hao et al., 2021). These results indicated that the whole cell catalysts from different sources have certain substrate specificity. In addition, whole cell catalysts from *Pseudomonas aeruginosa* displayed comparable catalytic activity to an immobilized lipase Novozyme 435 in synthesis of salidroside esters (Yang et al., 2021). From the results in Table 1, it was clear that salidroside was the appropriate substrate for the whole cell catalysts from *Aspergillus oryzae* and *Pseudomonas aeruginosa*.

**TABLE 1:** Regioselective caproylation of salidroside catalyzed by the whole cells.

Strains	V_0_ (mM/h)	*C* (%)	Regioselectivity (%)
*Rhizopus oryzae*	1.86 ± 0.09	20.83 ± 0.17	>99
*Aspergillus oryzae*	9.30 ± 0.36	95.72 ± 0.51	>99
*Pseudomonas aeruginosa*	8.92 ± 0.18	93.64 ± 0.35	>99
*Pseudomonas fluorescens*	1.21 ± 0.10	11.53 ± 0.13	>99
*Pseudomonas stutzeri*	0.82 ± 0.05	9.47 ± 0.06	>99

Reaction conditions: 0.04 mmol salidroside, 0.4 mmol vinyl caprylate, 0.02 g catalyst preparation, 2 ml anhydrous acetone, 40°C, 200 rpm.

High regioselectivity is one of the remarkable characteristics of biocatalysts. It is worth mentioning that all microbial whole-cell catalysts tested displayed absolute regioselectivities toward the 6′-hydroxyl of glucose in salidroside, which was comparable to the excellent selectivity of an immobilized lipase Novozyme 435 with salidroside 6′- caprylate being the sole ester product (Yang et al., 2021). Similarly, we recently found that *Aspergillus oryzae* whole cells exhibited excellent regioselectivities toward the primary hydroxyl group at allose moiety of helicid, a structural analogue of salidroside (Yang R. L. et al., 2020). The reason may be that the primary hydroxyl group has the less steric hindrance effect than the other hydroxyls. Based on the greater reaction efficiency and regioselectivity, *Aspergillus oryzae* strain was shortlisted for the subsequent investigation.

### 3.2 Optimization of salidroside ester synthesis

**FIGURE 1:**
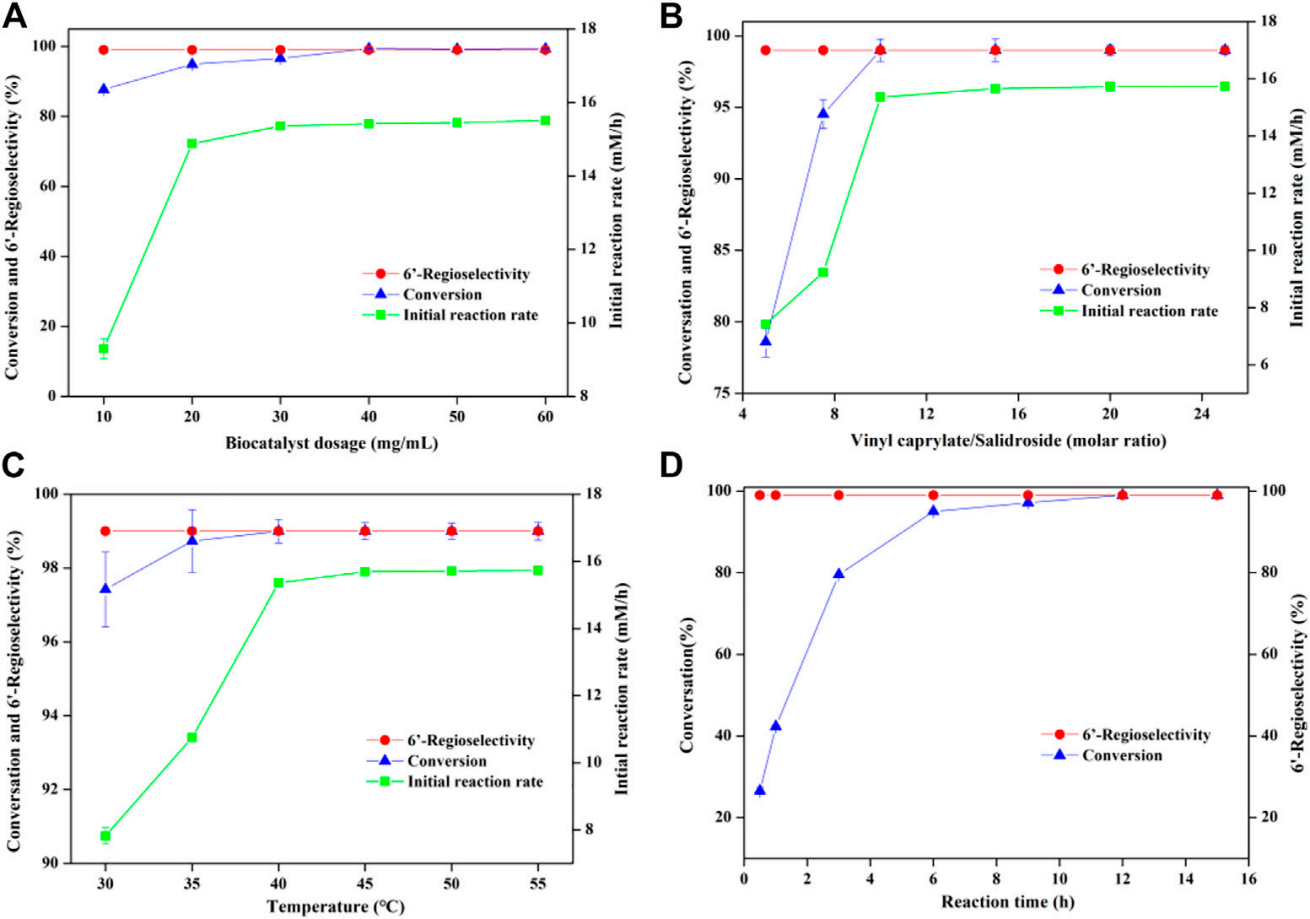
Regioselective caproylation of salidroside catalyzed by *Aspergillus oryzae* cells. **(A)** Effect of biocatalyst dosage [0.04 mmol salidroside, 0.4 mmol vinyl caprylate, 2 ml anhydrous acetone, 40°C, 200 rpm]. **(B)** Effect of molar ratio [0.04 mmol salidroside, 0.06 g. *Aspergillus oryzae* cells, 2 ml anhydrous acetone, 40°C, 200 rpm]. **(C)** Effect of temperature [0.04 mmol salidroside, 0.4 mmol vinyl caprylate, 0.06 g. *Aspergillus oryzae* cells, 2 ml anhydrous acetone, 200 rpm]. **(D)** Effect of reaction time [0.04 mmol salidroside, 0.4 mmol vinyl caprylate, 0.06 g. *Aspergillus oryzae* cells, 2 ml anhydrous acetone, 40°C, 200 rpm].

**FIGURE 2:**
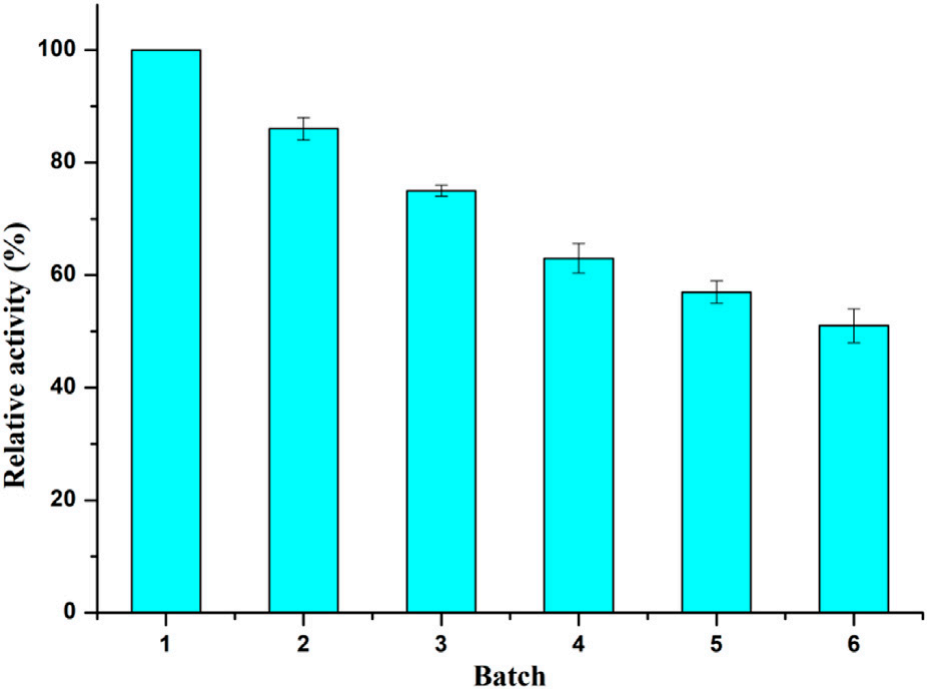
Operational stability of *Aspergillus oryzae* whole-cell catalyst. Reaction conditions: 0.04 mmol salidroside, 0.4 mmol vinyl caprylate, 0.06 g. *Aspergillus oryzae* cells, 2 ml anhydrous acetone, 40°C, 200 rpm.

In order to further improve the reaction efficiency, the synthesis of salidroside caprylate was used as a model reaction to investigate the influence of several key variables (reaction medium, molar ratio of substrate, whole-cell dosage) on the reaction. Like enzyme-mediated biotransformation, the nature of reaction medium has a significant effect on the whole-cell biocatalysis, which can impact the biocatalyst activity and stability. The presence of water may cause the hydrolysis of both the ester products and the acyl donors (vinyl esters), so several traditional organic solvents with different polarities from -0.23 to 1.85 were selected as reaction media (Table 2). Salidroside as a phenolic glycoside has high solubility in strongly polar solvents such as dimethyl sulfoxide (DMSO) and dimethylformamide (DMF) with 77.0 mM and 101.2 mM, respectively, while relatively low solubility in less polar organic solvents (25.3–42.1 mM). As speculated, *Aspergillus oryzae* has no catalytic activity in DMSO and DMF with strong polarity, which may inactivate lipases by destroying the membrane of whole-cell catalysts (Yang R. L. et al., 2019; Yang L. M. et al., 2020; Hao et al., 2021). Among the solvents tested (Figure 2), good conversions were obtained in 2-methyltetrahydrofuran (86.97%), tert-butanol (78.92%) and tetrahydrofuran (72.35%). And the highest conversion efficiency (95.72%) and initial reaction rate (9.30 mM/h) were obtained in acetone. Moreover, there was no significant correlation between the catalytic activity of the cells and the log P of the tested solvents, the commonly used solvent parameter in non-aqueous enzymology. As shown in Figure 1A, the reaction rate accelerated obviously (9.3 mM/h to 15.36 mM/h) with increasing the whole-cell dosage from 10 to 30 mg/ml, and then no obvious improvements occurred with further increasement of catalyst amount. The molar ratio of vinyl caprylate to salidroside had a great influence on the initial reaction rate and the maximal conversion (Figure 1B), which improved significantly with the increase of vinyl caprylate concentration up to 10 equivalents of salidroside concentration. A high conversion (>99%) and good initial reaction rate (15.36 mM/h) could be acquired with the molar ratio of vinyl caprylate to salidroside as 10. Figure 1C depicted that *Aspergillus oryzae* whole-cells had good biocatalysis performance in the temperature range of 30°C–55°C. Figure 1D showed the reaction process of salidroside acylation with vinyl caprylate under the above-obtained conditions. Salidroside conversion increased sharply at the initial stage of the reaction with 95% at 6 h, and then a slower rise, reaching 99% at 12 h.

**TABLE 2:** Effect of organic solvents on caproylation of salidroside catalyzed by *Aspergillus oryzae* cells.

Solvents	Lop	Solubility (mM)	V_0_ (mM/h)	*C* (%)	Regioselectivity (%)
Acetone	−0.23	164.3 ± 0.70	9.30 ± 0.22	95.72 ± 0.48	>99
Tetrahydrofuran	0.49	110.2 ± 0.61	2.05 ± 0.11	72.35 ± 0.29	>99
2-Methyltetrahydrofuran	1.85	20.6 ± 0.66	8.96 ± 0.15	86.97 ± 0.31	>99
*tert*-Butanol	0.60	31.5 ± 1.06	1.60 ± 0.08	78.92 ± 0.23	>99
DMSO	−1.3	632.6 ± 2.01	n.d.	n.d.	n.d.
DMF	−1.0	262.9 ± 1.15	n.d.	n.d.	n.d.

Reaction conditions: 0.04 mmol salidroside, 0.4 mmol vinyl caprylate, 0.02 g. *Aspergillus oryzae* cells, 2 ml anhydrous solvent, 40°C, 200 rpm. n.d., not detected.

Consider the above data comprehensively, the optimum conditions of reaction medium, *Aspergillus oryzae* amount, molar ratio of vinyl caprylate to salidroside and reaction temperature were acetone, 30 mg/ml, 10°C and 40°C, respectively, with initial rate of 15.36 mM/h and the highest substrate conversion of 99%. Additionally, the regioselectivity of the reaction maintained 99% under the above conditions.

### 3.3 Operational stability of the whole-cell biocatalyst

The reusability of biocatalyst is of great importance to the cost efficiency in industrial production. As shown in Figure 2, the *Aspergillus oryzae* whole cells remained 51% of its original activity after six consecutive batches. And the *Aspergillus oryzae* lost only 25% of its activity after three reaction cycles, which might be that the whole-cell provided a natural protective environment for intracellular enzyme with acylation activity.

### 3.4 Whole cell-mediated synthesis for aliphatic ester of salidroside

The synthesis of various salidroside aliphatic esters mediated by *Aspergillus oryzae* cells was explored in the above-mentioned optimal reaction conditions, with fatty acid vinyl esters of different chain lengths as acyl donors (Table 3). It is worth mentioning that 6′-monoesters of salidroside were exclusively achieved in all cases by NMR and HPLC analysis, which was similar to that in helicid esters synthesis catalyzed by *Aspergillus oryzae* cells, also affording an excellent selectivity for 6′-OH at allose moiety (Yang R. L. et al., 2020). However, in the synthesis of acetyl ester (a short alkyl acyl group) of salidroside by the commercial lipase Novozyme 435, 6′-O-acyl salidroside and 3′,6′-O-diacyl salidroside were the main two products, also with a small amount (<5%) of 2′,6′-O-diacryloyl salidroside (Yu et al., 2008). Thus *Aspergillus oryzae* cells exhibited superior regioselectivity to Novozyme 435 in the synthesis for short-chain aliphatic ester of salidroside, which highlighted the excellent regioselectivity of *Aspergillus oryzae* cells.

**TABLE 3:** Effect of various acyl donors on regioselective acylation of salidroside catalyzed by *Aspergillus oryzae* cells.

Acyl donor	Structural formula of acyl donors	V_0_ (mM/h)	Time (h)	*C* (%)	Regioselectivity (%)
Vinyl acetate	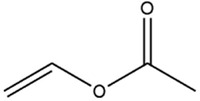	3.37 ± 0.09	24	96.1 ± 0.34	>99
Vinyl butyrate	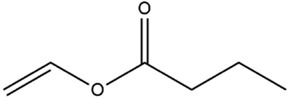	12.75 ± 0.11	12	97.4 ± 0.36	>99
Vinyl hexanoate	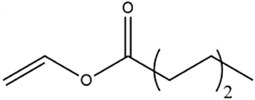	14.76 ± 0.25	12	99.0 ± 0.04	>99
Vinyl caprylate	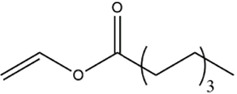	14.88 ± 0.17	12	99.0 ± 0.04	>99
Vinyl decanoate	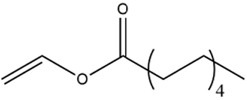	15.31 ± 0.20	12	99.0 ± 0.05	>99
Vinyl 10-undecenoate	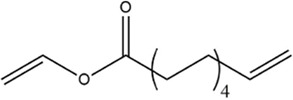	17.31 ± 0.19	12	99.0 ± 0.03	>99
Vinyl laurate	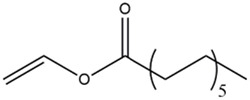	14.57 ± 0.21	12	98.5 ± 0.33	>99
Vinyl myristate	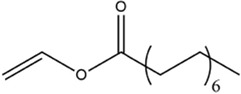	9.25 ± 0.13	12	97.3 ± 0.26	>99
Vinyl palmitate	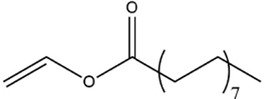	8.34 ± 0.12	12	96.5 ± 0.23	>99

Reaction conditions: 0.04 mmol salidroside, 0.4 mmol acyl donor, 0.06 g. *Aspergillus oryzae* cells, 2 ml anhydrous acetone, 40°C, 200 rpm.

As shown in Table 3, *Aspergillus oryzae* cells exhibited excellent catalytic activities with 96%–99% conversions in the salidroside acylation with different chain-length acyl donors. Furthermore, the initial reaction rate increased from 3.37 mM/h to 17.31 mM/h with the elongation of chain length of acyl donors from C2 to C11, which indicated that the interaction between medium-chain acyl groups and hydrophobic acyl binding sites of intracellular acylase was stronger than that of short-chain groups. Nevertheless, the initial reaction rate decreased with further extending chain length from C12 to C16, owing to the higher steric hindrance of the longer-chain acyl donors. Previous studies have found that the biocatalysts from different sources showed different catalytic properties for the different acyl donors due to the specific structure of the lipase active site and the acyl (Wang and Zong, 2009; Yang et al., 2013; Li X. F. et al., 2018; Yang W. et al., 2019). For example, the *Pseudomonas aeruginosa* cells were more specific toward the medium-chain acyl donors, as well as *Candida antarctica* lipase B (Novozyme 435) and *Thermomyces lanuginosus* lipase (Lipozyme TLL) (Wang and Zong, 2009; Yang et al., 2013; Yang R. L. et al., 2019), while the whole cells of *Candida parapsilosis* was most efficient for the short chain acyl donor, vinyl propionate. (Li G. Y. et al., 2018).

## 4 Conclusion

A facile and efficient biocatalytic approach was employed for the synthesis of salidroside esters by using *Aspergillus oryzae* cells, which was a potentially cost-attractive alternative to expensive immobilized enzymes. Various 6′-monoesters derivatives of salidroside were obtained with excellent conversions and high regioselectivities. And the structure of the acyl donors demonstrated an impact on the catalytic characteristic of the *Aspergillus oryzae* whole cells. The results afforded a green and highly efficient strategy for selective structural modification of polyhydroxyl natural products.

## Data Availability

The original contributions presented in the study are included in the article/Supplementary Material, further inquiries can be directed to the corresponding author.
