# Supplementary material for: A facile and efficient synthesis approach of salidroside esters by whole-cell biocatalysts in organic solvents
Source: Front Bioeng Biotechnol. 2022 Nov 24;10:1051117. doi: 10.3389/fbioe.2022.1051117 (PMC9729279; doi:10.3389/fbioe.2022.1051117)
Supplement: Supplementary file 1 [file DataSheet1.pdf]

Supplementary Information for

A facile and efficient synthesis approach of salidroside esters by whole-cell biocatalysts in organic solvents

Rongling Yang, Yu Wang, Xiangjie Zhao*, Zheng Tong, Qianlin Zhu, Xiaoxi He, Zhaoyu Wang, Hongzhen Luo, Fang Fang

School of Life Science and Food Engineering, Huaiyin Institute of Technology, Huaian 223003, China

*Corresponding author e-mail: zhaoxiangjie@hyit.edu.cn

Content

NMR data of salidroside and its esters..2

NMR spectra of salidroside and its esters..8

NMR data of salidroside and its esters

Salidroside:^{13}C NMR (100 MHz, DMSO-d_6): δ 35.31 (C_2), 61.58 ($C_{6'}$), 70.35 (C_1), 70.60 ($C_{4'}$), 73.93 ($C_{2'}$), 77.28 ($C_{3'}$), 77.35 ($C_{5'}$), 103.31 ($C_{1'}$), 115.49 (C_5+ C_7), 129.07 (C_3), 130.18 (C_4+ C_8), 156.09 (C_6).

^{1}H NMR (400 MHz, DMSO-d6): δ 2.81-2.67 (m, 2H, H_2), 2.96 (ddd, J = 8.9, 7.8, 4.9 Hz, 1H, $H_{4'}$), 3.20 – 3.00 (m, 3H, $H_{4'}$+ $H_{4'}$), 3.44 (dt, J = 11.6, 5.7 Hz, 1H, $H_{3'}$), 3.54-3.69 (m, 2H, H_1), 3.85-3.91 (m, 1H, $H_{2'}$), 4.18 (d, J = 7.8 Hz, 1H, $OH_{6'}$), 4.49 (t, *J* = 5.9 Hz, 1H, $H_{1'}$), 4.91 (d, *J* = 6.8 Hz, 1H, $OH_{3'}$), 4.94 (d, *J* = 4.0 Hz, 1H, $OH_{2'}$), 4.97 (d, *J* = 4.0 Hz, 1H, $OH_{4'}$), 6.69(d, J = 4.0 Hz, 2H, H_5+ H_7), 7.06 (d, J = 8.0 Hz, 2H, H_4+ H_8), 9.18 (s, 1H, OH_6).

Salidroside 6'-butyrate:^{13}C NMR (100 MHz, DMSO-d_6): δ 13.83 ($C_{4''}$), 14.53 ($C_{3''}$), 18.45 ($C_{2''}$), 35.87 (C_2), 60.20 ($C_{6'}$), 63.96 (C_1), 70.54 ($C_{4'}$), 73.79 ($C_{2'}$), 74.09 ($C_{3'}$), 76.95 ($C_{5'}$), 103.34 ($C_{1'}$), 115.46 (C_5+ C_7), 129.01 (C_3), 130.11 (C_4+ C_8), 156.07 (C_6), 173.11 ($C_{1''}$).

^{1}H NMR (400 MHz, DMSO-d6): δ 0.86 (t, J = 7.4 Hz, 3H, $H_{4''}$), 1.18 (t, J = 7.1 Hz, 2H, $H_{3''}$), 1.53 (h, J = 7.3 Hz, 2H, $H_{2''}$), 2.31-2.22 (m, 2H, H_2), 2.81-2.65 (m, 2H, H_1),3.23-2.92 (m, 3H, $H_{2'}$+ $H_{3'}$+ $H_{4'}$), 3.64-3.55 (m, 1H, $H_{6'}$), 3.86-3.76 (m, 1H, $H_{5'}$), 4.22 (d, J = 7.8 Hz, 1H, $H_{6'}$), 4.30 (dd, J = 11.8, 2.0 Hz, 1H, $OH_{3'}$), 5.05 (t, J = 6.5 Hz, 2H, $OH_{2'}$+ $OH_{4'}$), 5.16 (d, J = 5.4 Hz, 1H, $H_{1'}$), 6.73-6.60 (m, 2H, H_5+ H_7), 7.06-6.99 (m, 2H, H_4+ H_8), 9.16 (s, 1H, OH_6).

Salidroside 6'-hexanoate:^{13}C NMR (100 MHz, DMSO-d_6): δ 14.17 ($C_{6''}$), 22.17 ($C_{5''}$), 24.62 ($C_{4''}$), 31.04 ($C_{3''}$), 33.97 ($C_{2''}$), 35.36 (C_2), 60.21 ($C_{6'}$), 63.95 (C_1), 70.65

($C_{4'}$), 73.79 ($C_{2'}$), 74.10 ($C_{3'}$), 76.94 ($C_{5'}$), 103.36 ($C_{1'}$), 115.46 (C_5+ C_7), 128.99 (C_3), 130.10 (C_4+ C_8), 156.07 (C_6), 173.25 ($C_{1''}$).

^{1}H NMR (400 MHz, DMSO-d6): δ 0.82 (td, J = 5.5, 4.2, 2.1 Hz, 3H, $H_{6''}$), 1.20-1.25 (m, 4H, $H_{4''}$+ $H_{5''}$),1.44-1.55 (m, 2H, $H_{3''}$), 2.27 (t, J = 7.4 Hz, 2H, $H_{2''}$), 2.64-2.81 (m, 2H, H_2), 3.08 (td, J = 9.2, 5.4 Hz, 1H, $H_{4'}$), 3.18 (td, J = 8.8, 4.8 Hz, 1H, $H_{3'}$), 3.35 (ddd, J = 9.3, 6.8, 2.1 Hz, 2H, H_1), 3.54-3.65 (m, 1H, $H_{2'}$), 3.80 (ddd, J = 9.8, 8.4, 6.8 Hz, 1H, $H_{6'}$), 3.99-4.12 (m, 1H, $H_{5'}$), 4.22 (d, J = 7.8 Hz, 1H, $H_{6'}$), 4.30 (dd, J = 11.8, 2.0 Hz, 1H, $OH_{3'}$), 5.06 (dd, *J* = 8.9, 4.9 Hz, 2H, $OH_{2'}$+ $OH_{4'}$), 5.17 (d, *J* = 5.5 Hz, 1H, $H_{1'}$), 6.59-6.73(m , 2H, H_5+ H_7), 6.91-7.06 (m, 2H, H_4+ H_8), 9.18 (s, 1H, OH_6).

Salidroside 6'-caprylate:^{13}C NMR (100 MHz, DMSO-*d6*): *δ* 14.32 ($C_{8''}$), 22.47 ($C_{7''}$), 24.95 ($C_{3''}$), 28.76 ($C_{5''}$), 28.83 ($C_{4''}$), 31.52 ($C_{6''}$), 34.00 ($C_{2''}$), 35.39 (C_2), 60.19 ($C_{6'}$), 63.97 (C_1), 70.66 ($C_{4'}$), 73.79 ($C_{2'}$), 74.13 ($C_{3'}$), 76.96 ($C_{5'}$), 103.39 ($C_{1'}$), 115.46 (C_5+ C_7), 128.98 (C_3), 130.06 (C_4+ C_8), 156.09 (C_6), 173.21 ($C_{1''}$).

^{1}H NMR (400 MHz, DMSO-d6): δ 0.80 (t, J = 7.1 Hz, 3H, $H_{8''}$), 1.18 (s, 6H, $H_{6''}$+ $H_{5''}$+ $H_{7''}$),1.15-1.22 (m, 2H, $H_{4''}$), 1.36-1.44 (t, J = 7.4 Hz, 2H, $H_{3''}$), 2.18-2.26 (m, 2H, $H_{2''}$), 3.37-3.21 (m, 2H, H_2), 3.63 (ddd, *J* = 9.7, 7.3, 2.1 Hz, 1H, $H_{4'}$), 4.07 (dd, *J* = 11.8, 7.5 Hz, 1H, H_1), 4.38 – 4.23 (m, 1H, $H_{3'}$), 4.88 (d, *J* = 7.6 Hz, 1H, $H_{2'}$), 5.46 – 5.10 (m, 3H, $H_{6'}$+ $H_{5'}$), 6.31 (t, *J* = 2.2 Hz, 1H, $OH_{3'}$), 6.71-6.56 (m, 2H, $OH_{2'}$+ $OH_{3'}$), 6.76 (d, *J* = 8.1 Hz, 2H, $OH_{2'}$+ $OH_{4'}$), 6.86 (d, *J* = 16.3 Hz, 1H, H_5), 7.02(d, *J* = 16.3 Hz, 1H, H_7), 7.39 (d, 2H, H_4+ H_8), 9.56 (s, 1H, OH_6) .

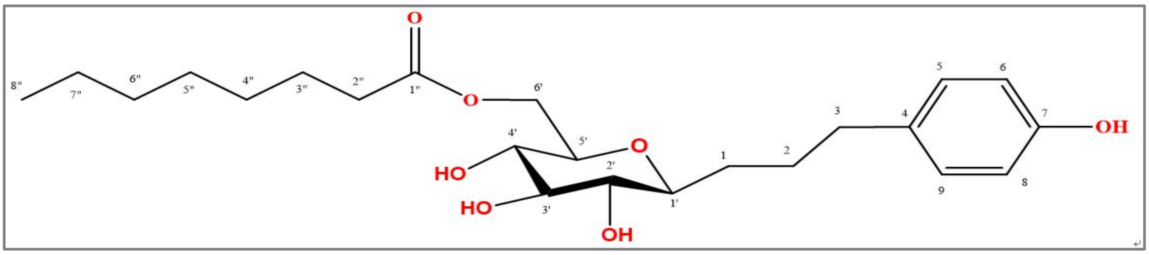

Supplementary Figure 1. Molecular structure of salidroside 6'-caprylate

Salidroside 6'-decanoate:^{13}C NMR (100 MHz, DMSO-d_6): δ 14.37 ($C_{10''}$), 22.55 ($C_{9''}$), 24.95 ($C_{3''}$), 28.88 ($C_{7''}$), 29.10 ($C_{5''}$), 29.12 ($C_{6''}$), 29.28 ($C_{4''}$), 31.73 ($C_{8''}$), 34.00 ($C_{2''}$), 35.39 (C_2), 60.18 ($C_{6'}$), 64.00 (C_1), 70.57 ($C_{4'}$), 73.79 ($C_{2'}$), 74.13 ($C_{3'}$), 76.96 ($C_{5'}$), 103.40 ($C_{1'}$), 115.45 (C_5+ C_7), 128.97 (C_3), 130.06 (C_4+ C_8), 156.10 (C_6), 173.21 ($C_{1''}$).

^{1}H NMR (400 MHz, DMSO-d_6): δ 0.85 (t, J = 6.8 Hz, 3H, $H_{10''}$), 1.21 (q, J = 5.5, 4.6 Hz, 12H, $H_{4''}$+ $H_{5''}$ +$H_{6''}$+ $H_{7''}$ +$H_{8''}$+ $H_{9''}$), 1.50 (p, J = 7.2 Hz, 2H, $H_{3''}$), 2.27 (t, J = 7.3 Hz, 2H, $H_{2''}$), 2.74 (tt, J = 9.4, 6.8 Hz, 2H, H_2), 2.99 (td, J = 8.4, 3.3 Hz, 1H, $H_{4'}$), 3.08 (t, J = 9.0 Hz, 1H, $H_{3'}$), 3.18 (t, J = 8.9 Hz, 1H, H_1), 3.35 (ddd, J = 9.3, 6.9, 2.1 Hz, 1H, H_1), 3.60 (ddd, J = 9.8, 8.3, 6.7 Hz, 1H, $H_{2'}$), 3.81 (ddd, J = 9.8, 8.2, 6.8 Hz, 1H, $H_{6'}$), 4.03-4.10 (m, 1H, $H_{5'}$), 4.21 (d, J = 7.8 Hz, 1H, $H_{6'}$), 4.30 (dd, J = 11.7, 2.0 Hz, 1H, $H_{1'}$), 4.91-5.22 (m, 3H, $OH_{2'}$+ $OH_{3'}$ + $OH_{4'}$), 6.60-6.74 (m, 2H, H_5+ H_7), 6.98-7.09 (m, 2H, H_4+ H_8), 9.14 (s, 1H, OH_6).

Salidroside 10-undecenoate:^{13}C NMR (100 MHz, DMSO-d_6): δ 24.95 ($C_{3''}$), 28.73 ($C_{5''}$), 28.87 ($C_{4''}$), 28.93 ($C_{8''}$), 29.08 ($C_{7''}$), 29.14 ($C_{6''}$), 33.63 ($C_{9''}$), 34.01 ($C_{2''}$), 35.40 (C_2), 64.00 ($C_{6'}$), 70.58 (C_1), 70.67 ($C_{4'}$), 73.79 ($C_{2'}$), 74.14 ($C_{3'}$), 76.97 ($C_{5'}$), 103.40 ($C_{1'}$), 114.98 ($C_{11'}$), 115.46 (C_5+ C_7), 128.97 (C_3), 130.06 (C_4+ C_8), 173.23 ($C_{10''}$), 156.10 (C_6), 173.19 ($C_{1''}$).

^{1}H NMR (400 MHz, DMSO-d_6): δ 1.23 (dd, J = 11.1, 4.3 Hz, 8H, $H_{5''}$ +$H_{6''}$+ $H_{7''}$ +$H_{8''}$), 1.32 (p, J = 6.9 Hz, 2H, $H_{7''}$), 1.50 (p, J = 7.0 Hz, 2H, $H_{4''}$), 2.00 (q, J = 7.0 Hz, 2H, $H_{9''}$), 2.27 (t, J = 7.3 Hz, 2H, $H_{2''}$), 2.74 (td, J = 7.3, 2.7 Hz, 2H, H_2), 3.00 (td, J = 8.4, 4.7 Hz, 1H, $H_{4'}$), 3.09 (td, J = 9.2, 5.3 Hz, 1H, H_1), 3.19 (td, J = 8.8, 4.5 Hz, 1H, H_1), 3.36 (ddd, J = 9.3, 6.9, 2.1 Hz, 1H, $H_{3'}$), 3.55-3.65 (m, 1H, $H_{2'}$),3.82 (dt, J = 10.0, 7.4 Hz, 1H, $H_{6'}$), 4.08 (dd, J = 11.7, 6.9 Hz,1H, $H_{5'}$), 4.22 (d, J = 7.7 Hz, 1H, $H_{6'}$), 4.31 (dd, J = 11.8, 2.1 Hz, 1H, $OH_{3'}$), 4.89-5.10 (m, 4H, $H_{11''}$+ $OH_{2'}$ + $OH_{4'}$), 5.15 (d, J = 5.4 Hz, 1H, $H_{1'}$), 5.78 (ddt, J = 16.9, 10.1, 6.6 Hz, 1H, $H_{10''}$), 6.62-6.72 (m, 2H, H_5+ H_7), 7.02 (d, J = 8.2 Hz, 2H, H_4+ H_8), 9.13 (s, 1H, OH_6).

Salidroside 6'-laurate:^{13}C NMR (100 MHz, DMSO-d_6): δ 14.35 ($C_{12''}$), 22.56 ($C_{11''}$), 24.95 ($C_{3''}$), 28.90 ($C_{4''}$), 29.15 ($C_{5''}$), 29.19 ($C_{9''}$), 29.34 ($C_{6''}$), 29.48 ($C_{8''}$+$C_{7''}$), 31.77 ($C_{10''}$), 34.00 ($C_{2''}$), 35.40 (C_2), 60.16 ($C_{6'}$), 64.01 (C_1), 70.56 ($C_{4'}$), 73.79 ($C_{2'}$), 74.15 ($C_{3'}$), 76.97 ($C_{5'}$), 103.40 ($C_{1'}$), 115.44 (C_5+ C_7), 128.96 (C_3), 130.03 (C_4+ C_8), 156.11 (C_6), 173.16 ($C_{1''}$).

^{1}H NMR (400 MHz, DMSO-d_6): δ 0.86 (t, J = 6.7 Hz, 3H, $H_{12''}$), 1.19-1.30 (m, 16H, $H_{4''}$ +$H_{5''}$ +$H_{6''}$+ $H_{7''}$ +$H_{8''}$+ $H_{9''}$+ $H_{10''}$+ $H_{11''}$), 1.50 (p, J = 7.2 Hz, 2H, $H_{3''}$), 2.27 (t, J = 7.4 Hz, 2H, $H_{2''}$), 2.74 (ddd, J = 8.8, 6.9, 2.4 Hz, 2H, H_2), 3.01 (td, J = 8.4, 4.9 Hz, 1H, $H_{4'}$), 3.09 (td, J = 9.2, 5.4 Hz, 1H, H_1), 3.19 (td, J = 8.8, 4.7 Hz, 1H, H_1), 3.36 (ddd, J = 9.3, 6.9, 2.1 Hz, 2H, $H_{3'}$), 3.60 (ddd, J = 9.8, 8.2, 6.7 Hz, 1H, $H_{2'}$), 3.82 (dt, J = 9.8, 7.3 Hz, 1H, $H_{6'}$), 4.01-4.12 (m, 1H, H_5), 4.22 (d, J = 7.7 Hz, 1H, $H_{6'}$), 4.31 (dd, J = 11.8, 2.0 Hz, 1H, $OH_{3'}$), 5.05 (dd, J = 4.9, 3.4 Hz, 2H, $OH_{2'}$+ $OH_{4'}$), 5.15 (d, J = 5.4 Hz, 1H, $H_{1'}$), 6.62-6.71 (m, 2H, H_5+ H_7), 6.98-7.05 (m, 2H, H_4+ H_8), 9.12 (s, 1H,

OH_6).

Salidroside 6'-myristate:^{13}C NMR (100 MHz, DMSO-*d6*): *δ* 14.33 ($C_{14''}$), 22.57 ($C_{13''}$), 24.96 ($C_{3''}$), 28.93 ($C_{4''}$), 29.17 ($C_{5''}$), 29.21 ($C_{11''}$), 29.37 ($C_{10''}$), 29.51 ($C_{6''}$), 29.52 ($C_{9''}$), 29.53 ($C_{8''}$), 29.56 ($C_{7''}$), 31.79 ($C_{12''}$), 33.99 ($C_{2''}$), 35.41 (C_2), 60.16 ($C_{6'}$), 64.03 (C_1), 70.55 ($C_{4'}$), 73.78 ($C_{2'}$), 74.15 ($C_{3'}$), 76.97 ($C_{5'}$), 103.41 ($C_{1'}$), 115.44 (C_5+ C_7), 128.95 (C_3), 130.02 (C_4+ C_8), 156.12 (C_6), 173.14 ($C_{1''}$).

^{1}H NMR (400 MHz, DMSO-*d6*): δ 0.79-0.91 (m, 3H, $H_{14''}$), 1.22 (d, *J* = 9.4 Hz, 20H, $H_{4''}$ +$H_{5''}$ +$H_{6''}$+ $H_{7''}$ +$H_{8''}$+ $H_{9''}$+ $H_{10''}$+ $H_{11''}$+ $H_{12''}$+ $H_{13''}$), 1.50 (p, *J* = 7.1 Hz, 2H, $H_{3''}$), 2.27 (t, *J* = 7.4 Hz, 2H, $H_{2''}$), 2.74 (td, *J* = 7.3, 2.5 Hz, 2H, H_2), 3.01 (t, *J* = 8.4 Hz, 1H, $H_{4'}$), 3.09 (t, *J* = 9.2 Hz, 1H, H_1), 3.19 (t, *J* = 8.8 Hz, 1H, H_1), 3.36 (ddd, *J* = 9.3, 6.9, 2.1 Hz, 1H, $H_{3'}$), 3.60 (dt, *J* = 9.5, 7.4 Hz, 1H, $H_{2'}$), 3.82 (dt, *J* = 10.0, 7.4 Hz, 1H, $H_{6'}$), 3.99-4.14 (m, 1H, $H_{5'}$), 4.22 (d, *J* = 7.8 Hz, 1H, $H_{6'}$), 4.31 (dd, *J* = 11.8, 2.0 Hz, 1H, $H_{1'}$), 5.07 (s, 3H, $OH_{2'}$+ $OH_{3'}$+ $OH_{4'}$) 6.60-6.70 (m, 2H, H_5+ H_7), 6.95-7.07 (m, 2H, H_4+ H_8), 9.12 (s, 1H, OH_6).

Salidroside 6'-palmitate:^{13}C NMR (100 MHz, DMSO-*d6*): *δ* 14.37 ($C_{16''}$), 22.55 ($C_{15''}$), 24.95 ($C_{3''}$), 28.89 ($C_{5''}$), 29.13 ($C_{13''}$), 29.17 ($C_{4''}$), 29.32 ($C_{11''}$), 29.45 ($C_{12''}$), 29.48 ($C_{8''}$+$C_{7''}$), 29.51 ($C_{6''}$+$C_{9''}$+$C_{10''}$), 31.76 ($C_{14''}$), 34.00 ($C_{2''}$), 35.39 (C_2), 64.01 ($C_{6'}$), 70.55 (C_1), 70.66 ($C_{4'}$), 73.79 ($C_{2'}$), 74.13 ($C_{3'}$), 76.96 ($C_{5'}$), 103.39 ($C_{1'}$), 115.44 (C_5+ C_7), 128.96 (C_3), 130.06 (C_4+ C_8), 156.10 (C_6), 173.20 ($C_{1''}$).

^{1}H NMR (400 MHz, DMSO-*d6*): δ 0.81-0.92 (m, 3H, $H_{16''}$), 1.22 (d, *J* = 12.7 Hz, 24H, $H_{4''}$ +$H_{5''}$ +$H_{6''}$+ $H_{7''}$ +$H_{8''}$+ $H_{9''}$+ $H_{10''}$+ $H_{11''}$+ $H_{12''}$+ $H_{13''}$+ $H_{14''}$+ $H_{15''}$), 1.50 (p, *J* = 7.2 Hz, 2H, $H_{3''}$), 2.27 (t, *J* = 7.4 Hz, 2H, $H_{2''}$), 2.73 (tt, *J* = 9.3, 6.8 Hz, 2H, H_2), 2.98

(td, J = 8.3, 4.9 Hz, 1H, $H_{4'}$), 3.07 (td, J = 9.2, 5.4 Hz, 1H, H_1), 3.17 (td, J = 8.8, 4.8 Hz, 1H, H_1), 3.32-3.36 (m, 1H, $H_{3'}$), 3.59 (ddd, J = 9.8, 8.3, 6.7 Hz, 1H, $H_{2'}$), 3.80 (dt, J = 9.8, 7.3 Hz, 1H, $H_{6'}$), 4.06 (dd, J = 11.8, 6.9 Hz, 1H, $H_{5'}$), 4.21 (d, J = 7.8 Hz, 1H, $H_{6'}$), 4.30 (dd, J = 11.8, 2.0 Hz, 1H, $OH_{3'}$), 5.05 (dd, J = 6.2, 4.9 Hz, 2H, $OH_{2'}$+ $OH_{4'}$)), 5.16 (d, J = 5.4 Hz, 1H, $H_{1'}$), 6.62-6.70 (m, 2H, H_5+ H_7), 7.06 – 6.96 (m, 2H, H_4+ H_8), 9.14 (s, 1H, OH_6).

NMR spectra of salidroside and its esters

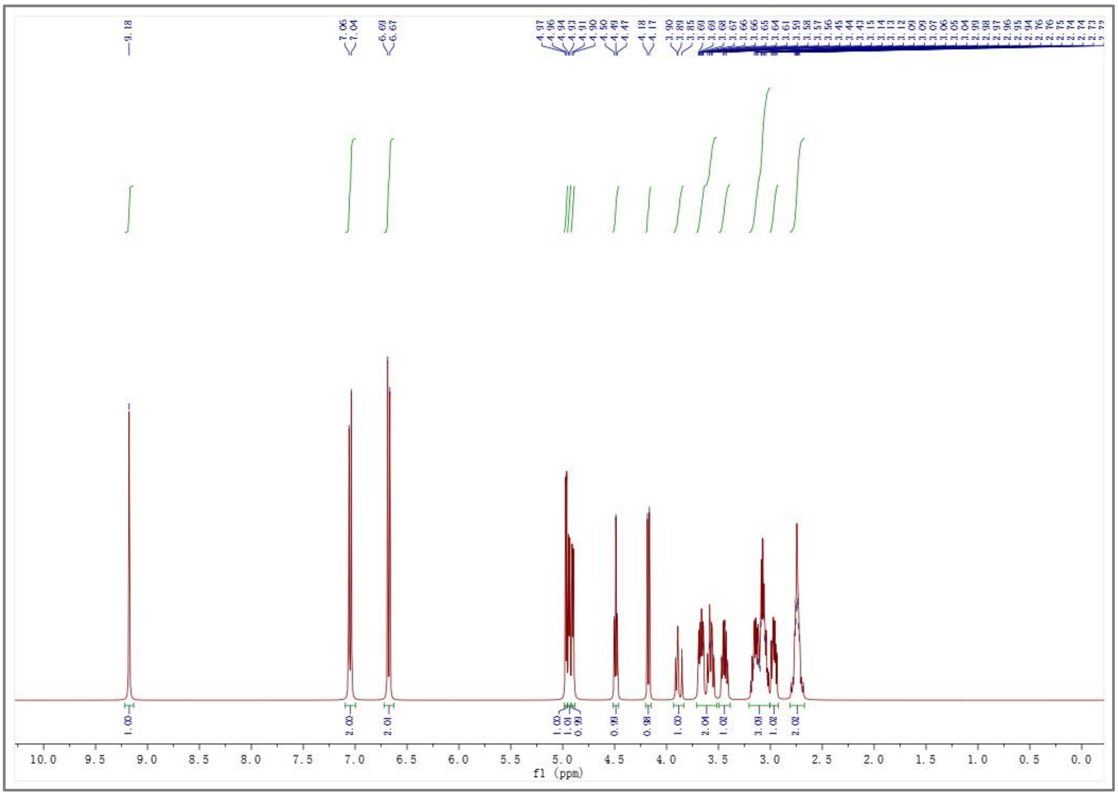

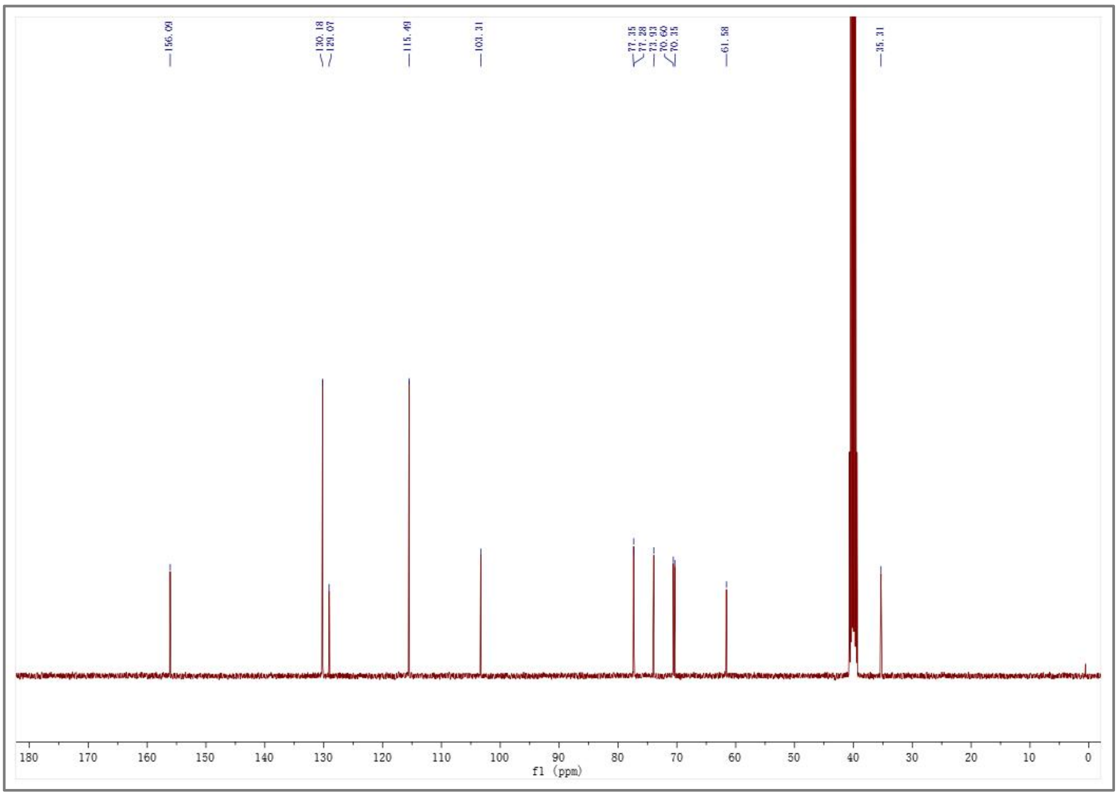

Supplementary Figure 2. NMR spectra of salidroside (^{1}H NMR in DMSO-d_6), up; (^{13}C NMR in DMSO-d_6), down

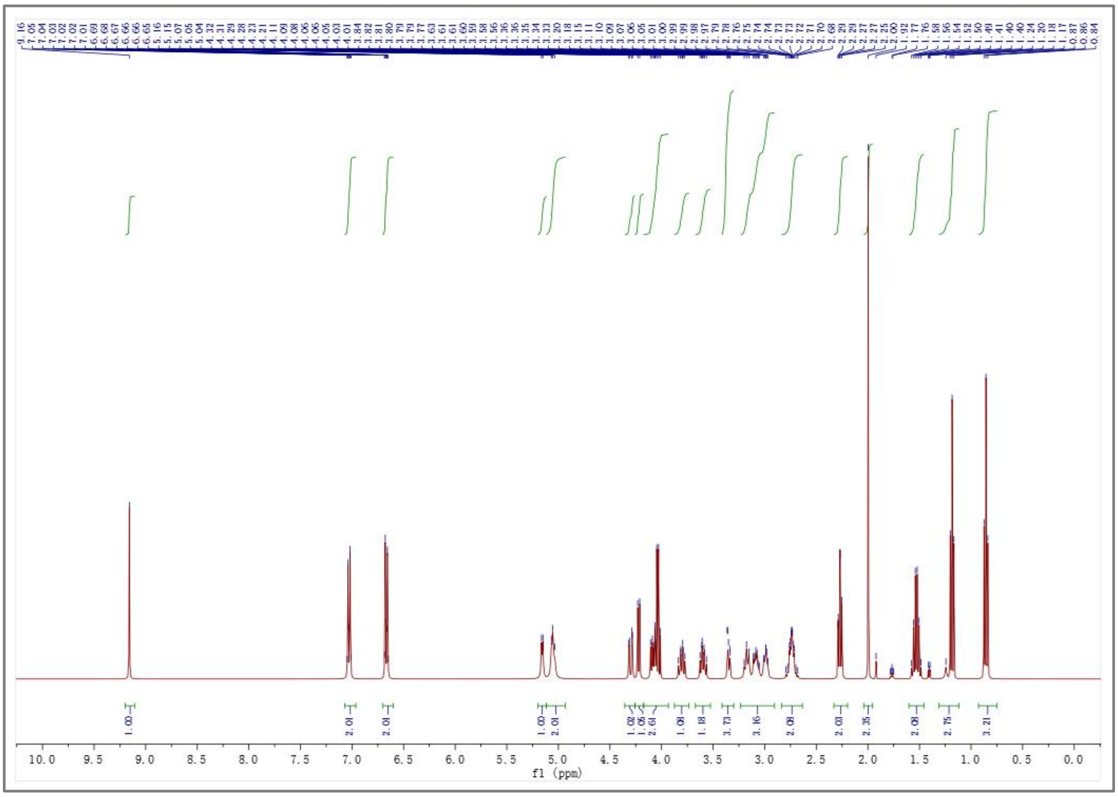

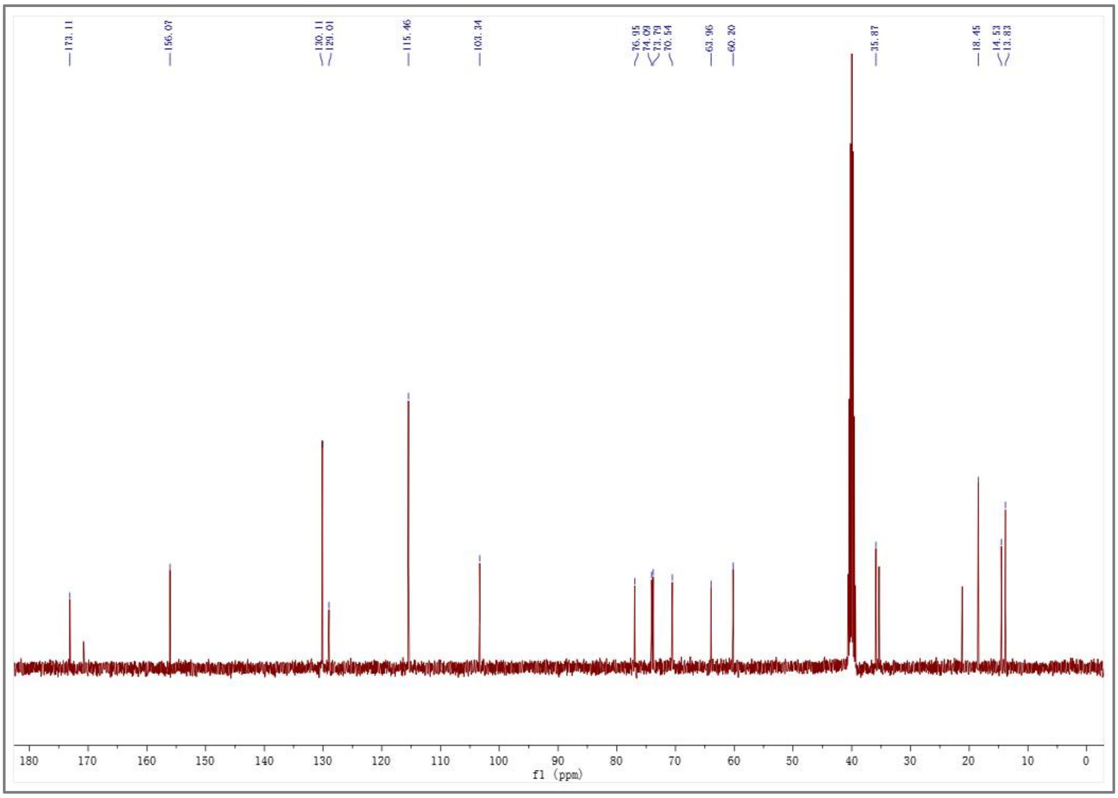

Supplementary Figure 3. NMR spectra of salidroside 6'-butyrate (^{1}H NMR in DMSO-d_6), up; (^{13}C NMR in DMSO-d_6), down

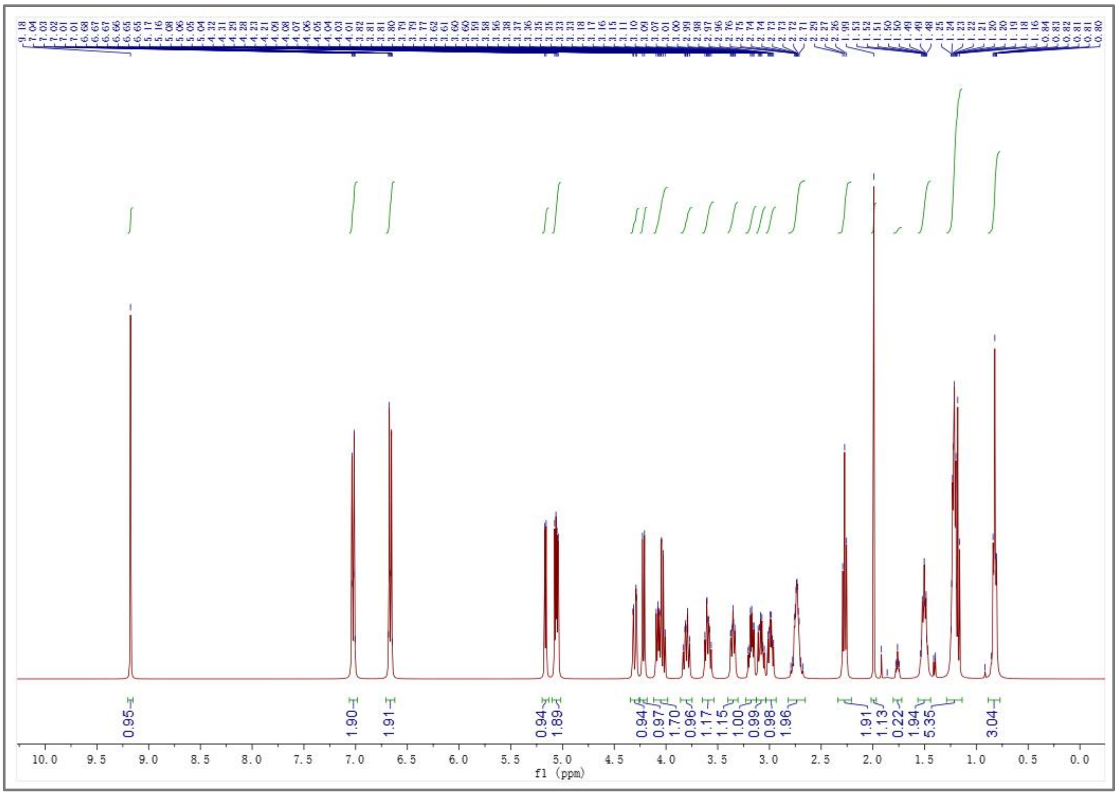

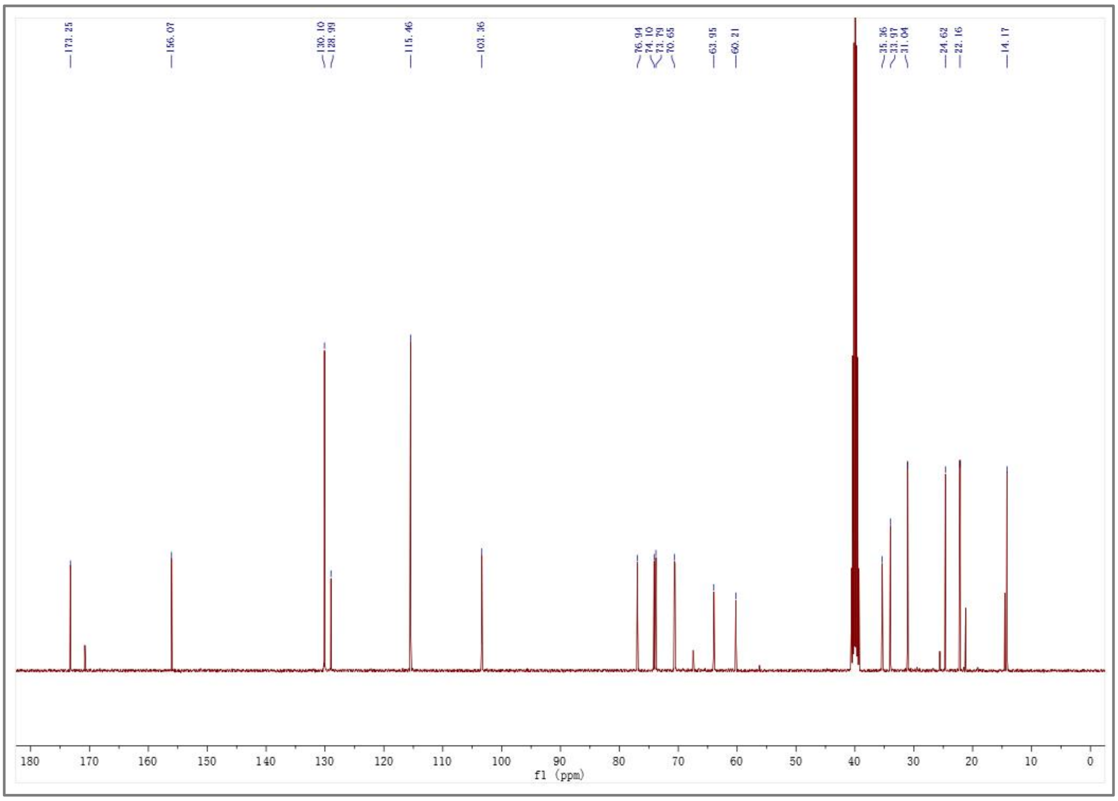

Supplementary Figure 4. NMR spectra of salidroside 6'-hexanoate (^{1}H NMR in DMSO-d_6), up; (^{13}C NMR in DMSO-d_6), down

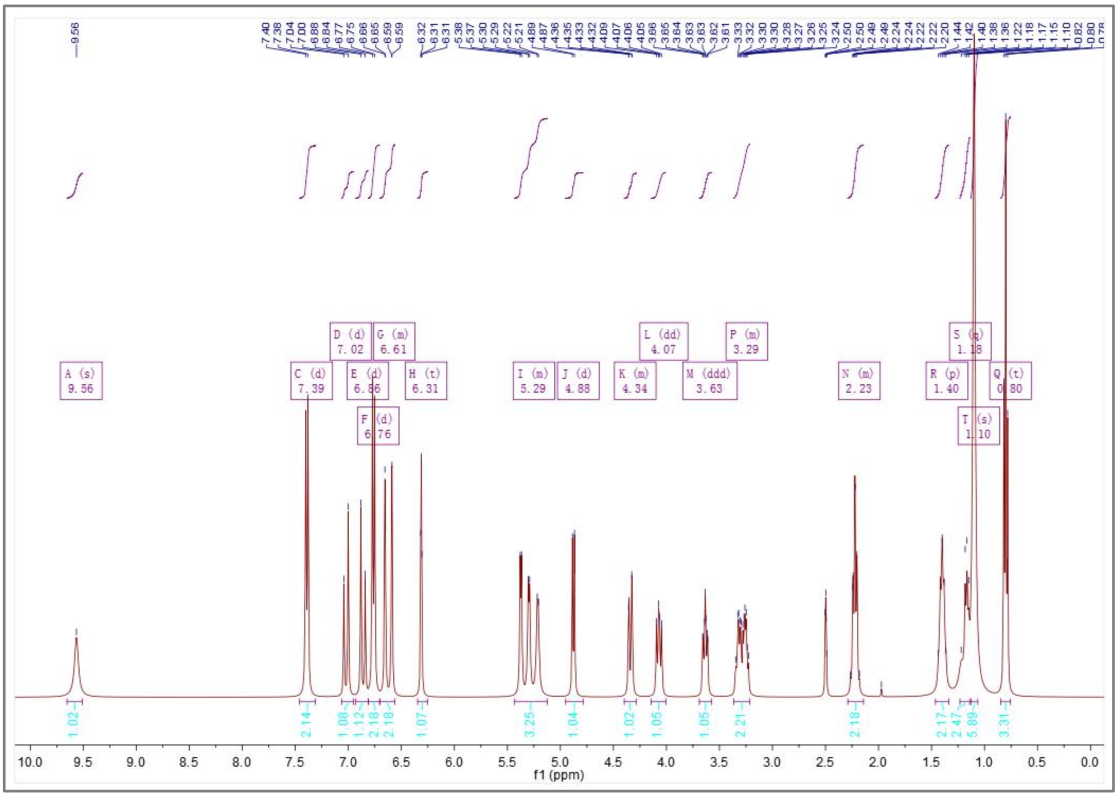

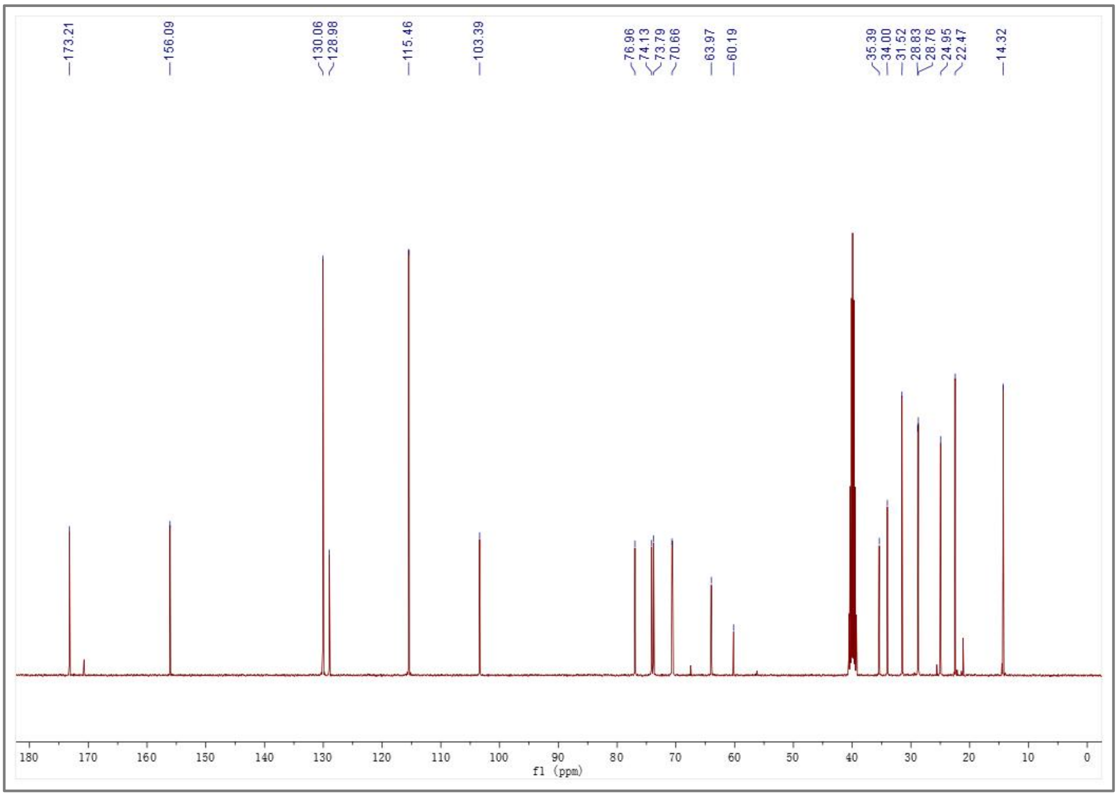

Supplementary Figure 5. NMR spectra of salidroside 6'-caprylate (^{1}H NMR in DMSO-d_6), up; (^{13}C NMR in DMSO-d_6), down

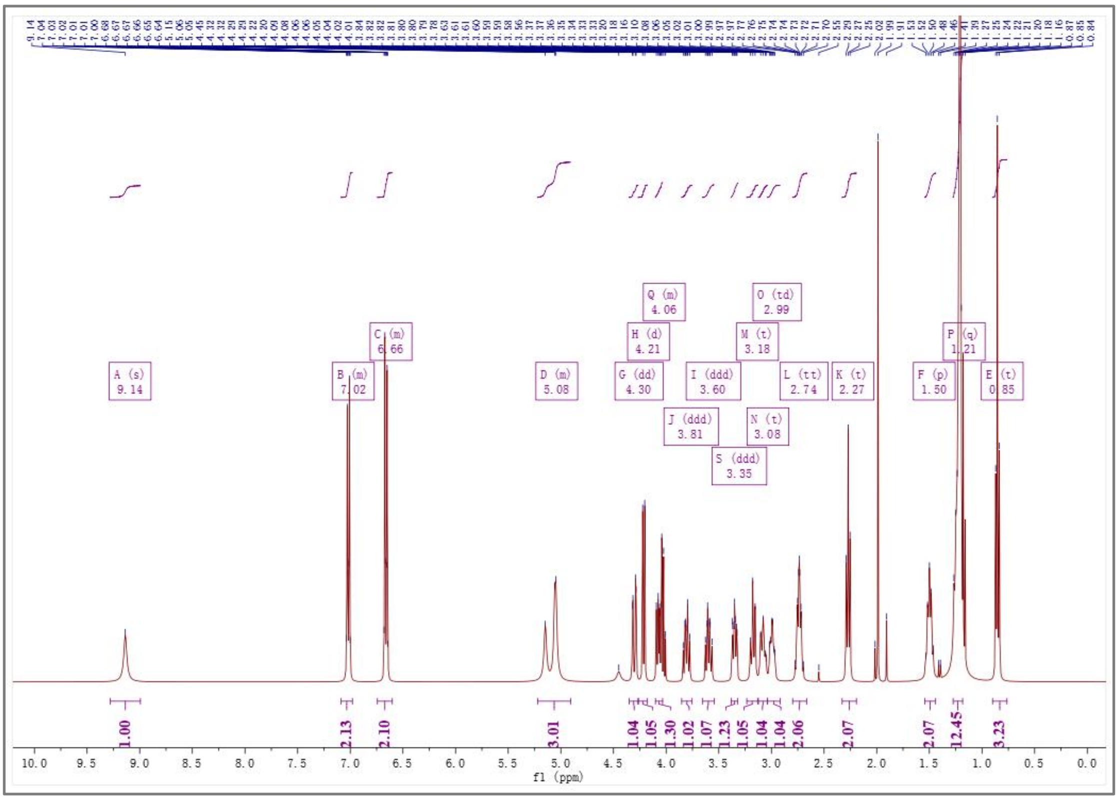

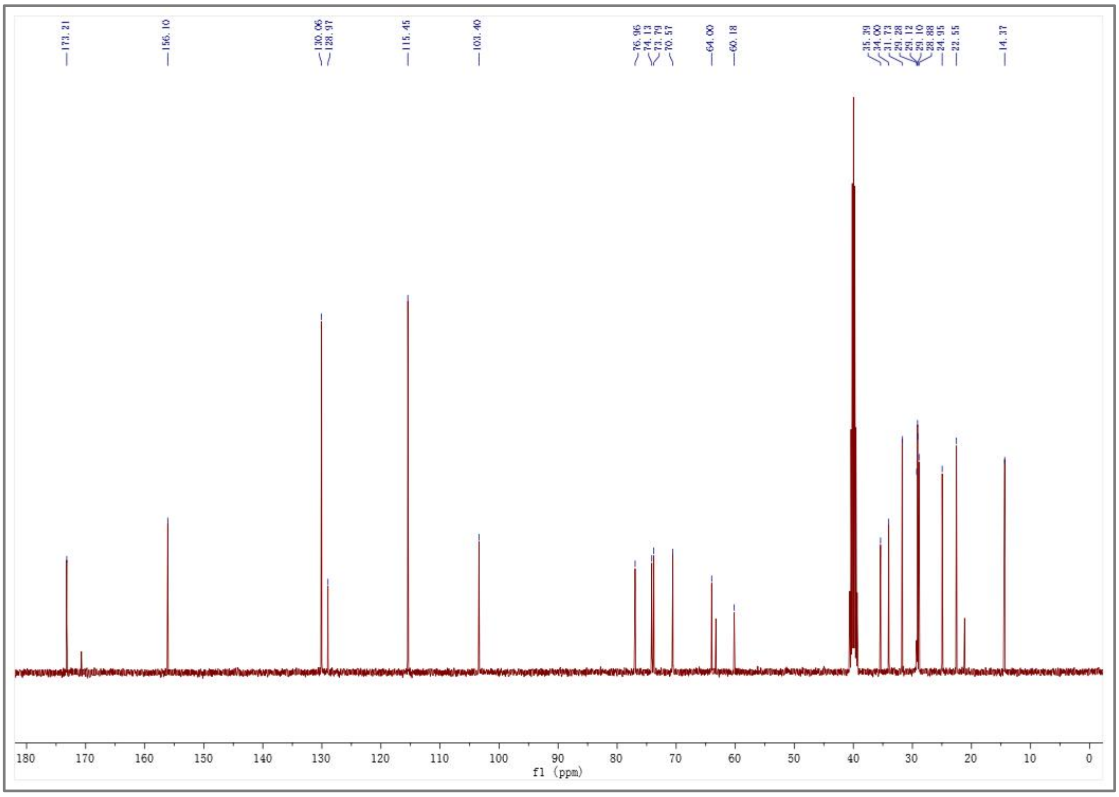

Supplementary Figure 6. NMR spectra of salidroside 6'-decanoate (^{1}H NMR in DMSO-d_6), up; (^{13}C NMR in DMSO-d_6), down

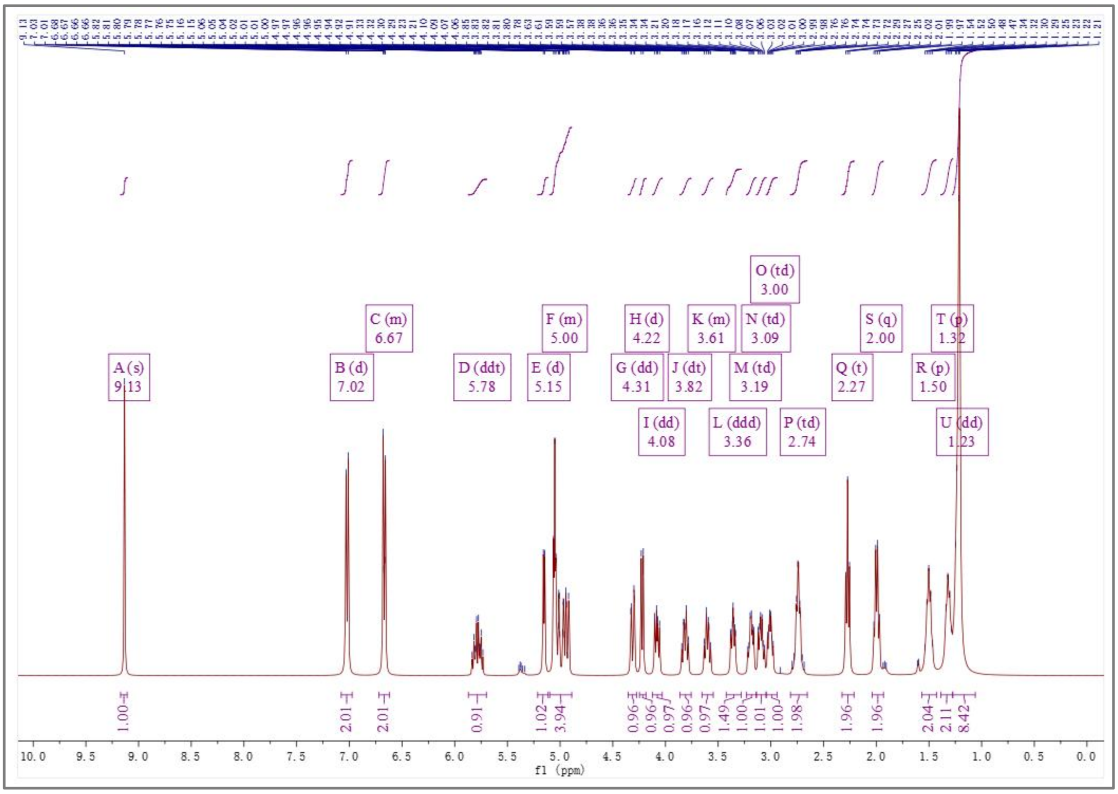

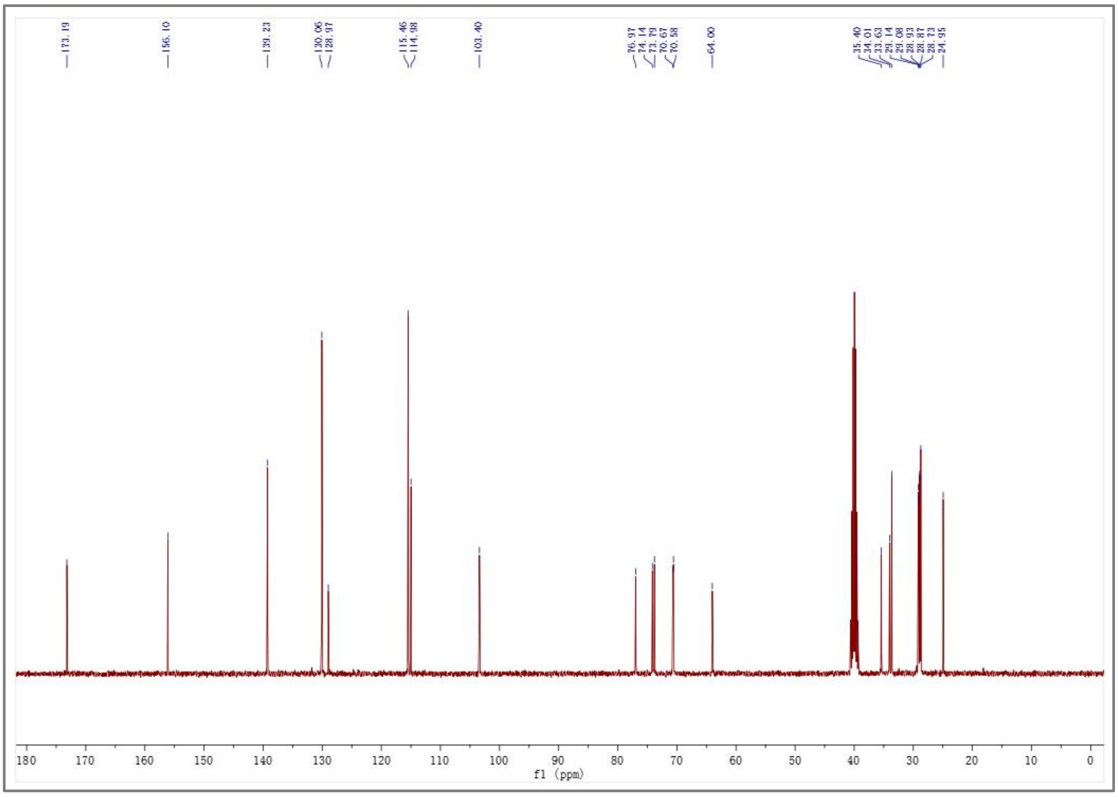

Supplementary Figure 7. NMR spectra of salidroside 10-undecenoate (^{1}H NMR in DMSO-d_6), up; (^{13}C NMR in DMSO-d_6), down

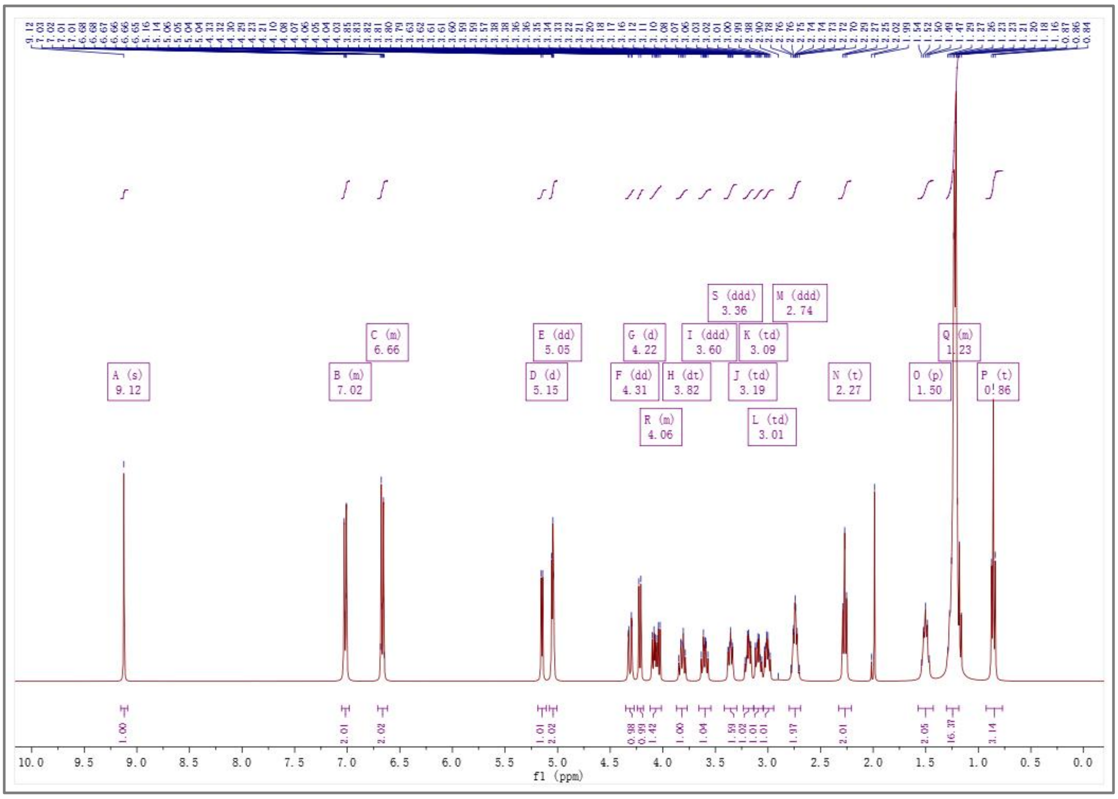

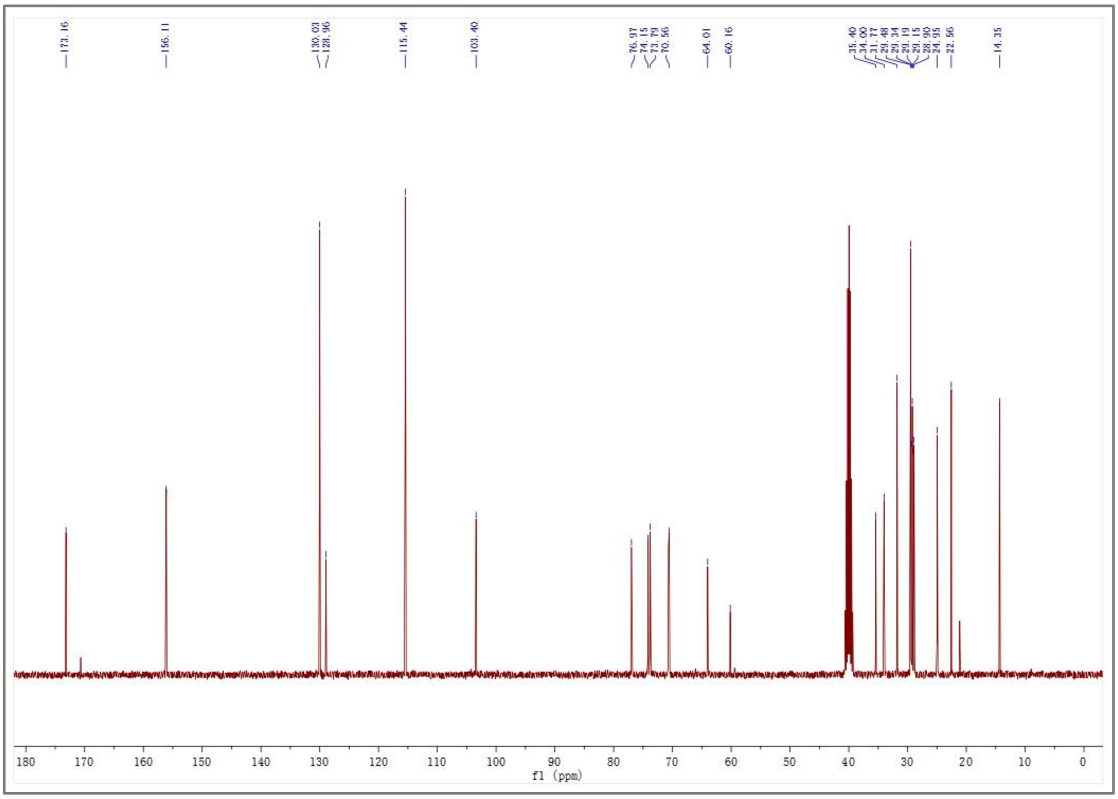

Supplementary Figure 8. NMR spectra of salidroside 6'-laurate (^{1}H NMR in DMSO-d_6), up; (^{13}C NMR in DMSO-d_6), down

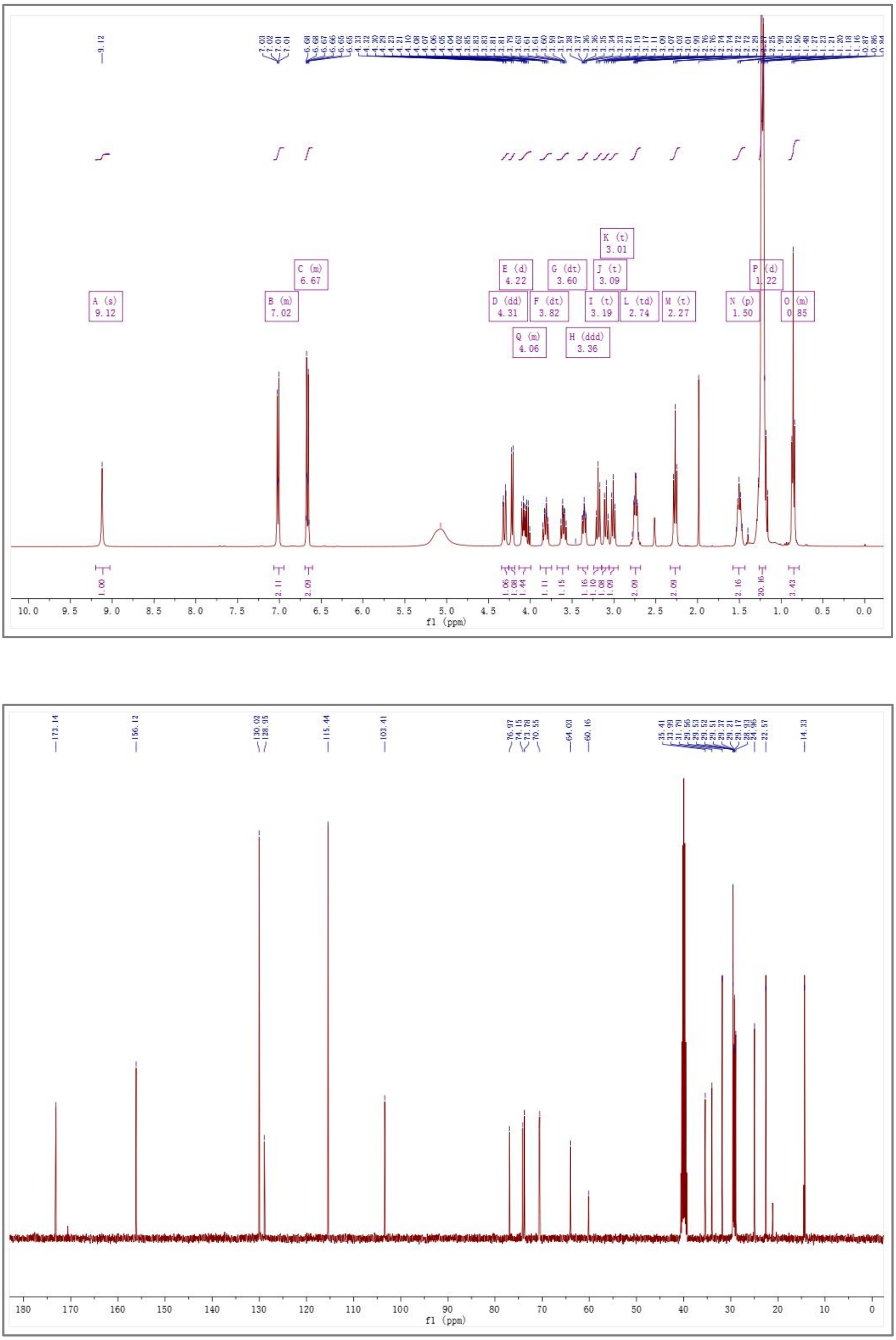

Supplementary Figure 9. NMR spectra of salidroside 6'-myristate (^{1}H NMR in DMSO-d_6), up; (^{13}C NMR in DMSO-d_6), down

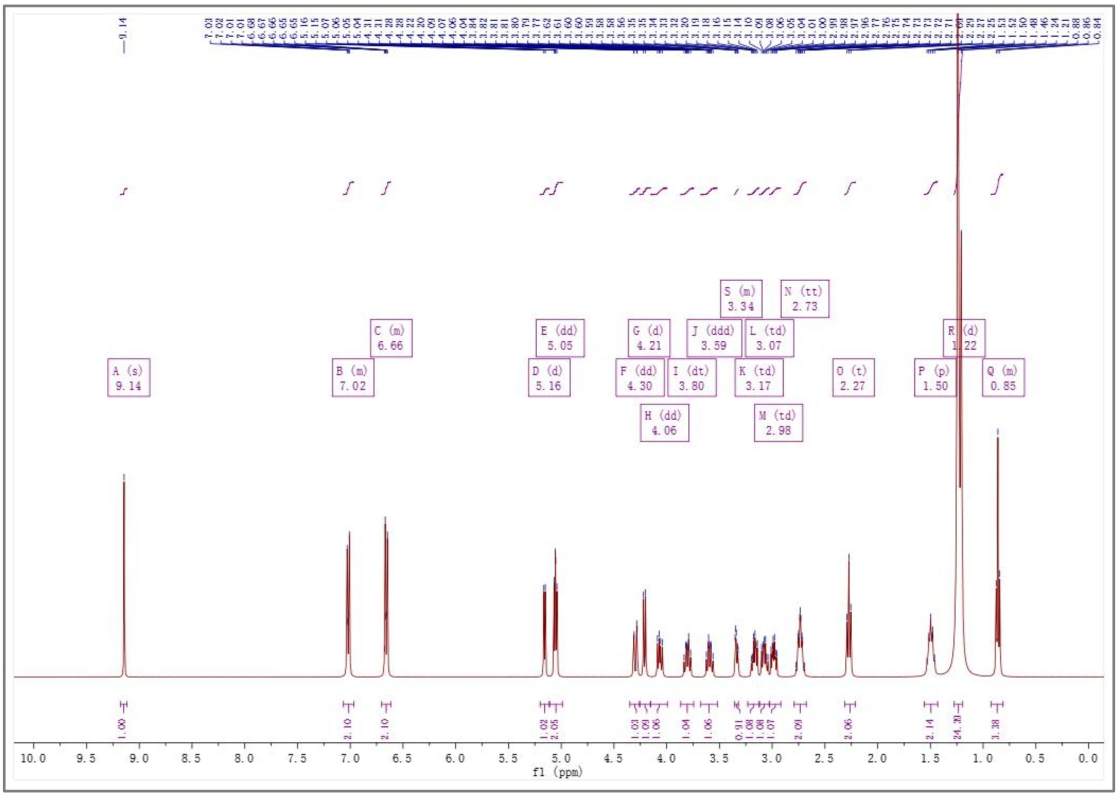

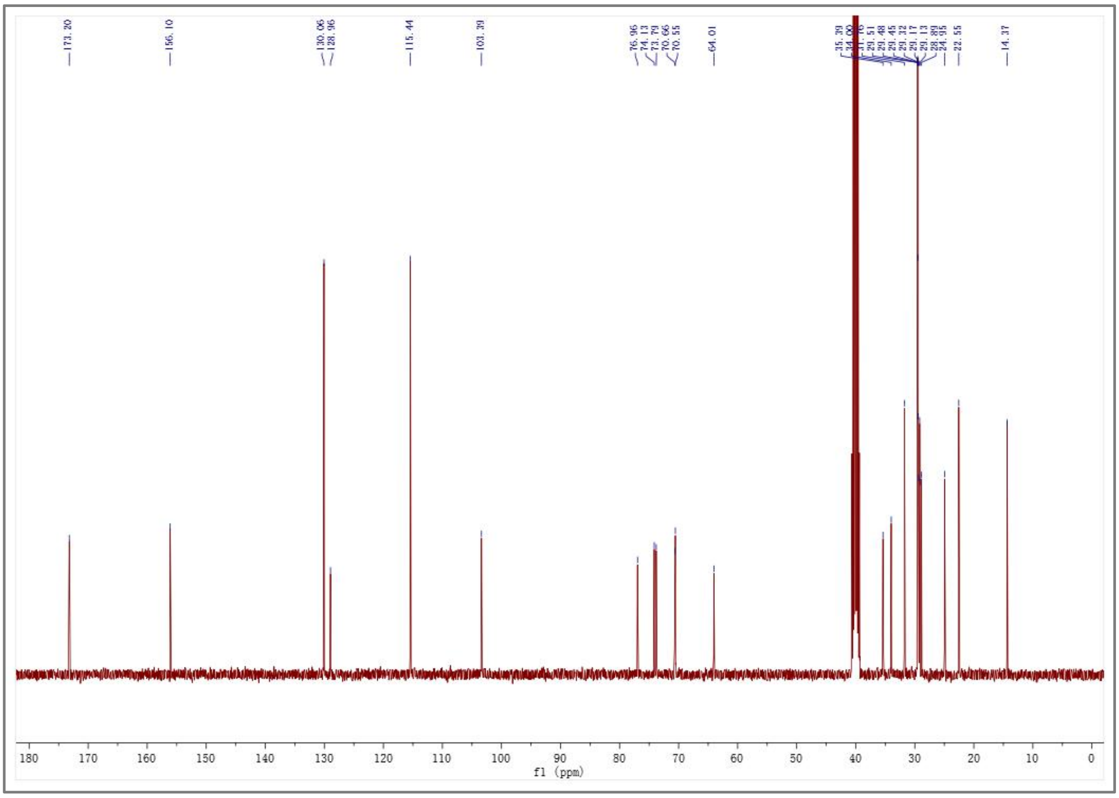

Supplementary Figure 10. NMR spectra of salidroside 6'-palmitate (^{1}H NMR in DMSO-d_6), up; (^{13}C NMR in DMSO-d_6), down
